# Short Glass Fiber-Reinforced Recycled Polyethylene Terephthalate Composites for Additive Manufacturing: Modification Strategies, Processing, Characterization and 3D Printing

**DOI:** 10.3390/polym18101155

**Published:** 2026-05-08

**Authors:** Izabela Irska, Mateusz Kasprowiak, Piotr Franciszczak, Sandra Paszkiewicz, Katarzyna Gawdzińska, Elżbieta Piesowicz

**Affiliations:** 1 Faculty of Mechanical Engineering and Mechatronics, West Pomeranian University of Technology in Szczecin, 70-310 Szczecin, Poland; sandra.paszkiewicz@zut.edu.pl (S.P.); elzbieta.senderek@zut.edu.pl (E.P.); 2 PPHU POLIGRAF Wiesław Kasprowiak, Zielona Street 33, 66-400 Gorzów Wielkopolski, Poland; m.kasprowiak@papierdoplotera.com; 3 Department of Machines Construction and Materials, Faculty of Marine Engineering, Maritime University of Szczecin, Willowa Street 2, 71-650 Szczecin, Poland; p.franciszczak@pm.szczecin.pl (P.F.); k.gawdzinska@pm.szczecin.pl (K.G.)

**Keywords:** recycling, polyethylene terephthalate, 3D printing, additive manufacturing, composites, glass fiber reinforcement

## Abstract

In response to the growing demand for sustainable manufacturing, 3D printing using recycled polyethylene terephthalate (rPET) offers a novel waste-to-value conversion method. Although the application of rPET in additive manufacturing has attracted significant attention from both the academic and industrial sectors, substantial challenges impede its further development, notably the high processing shrinkage and poor mechanical properties of the final product. This study focuses on developing recycled PET-based composites with favorable processing, thermal, and mechanical properties. Regranulates were produced via twin-screw extrusion using PET flakes, multifunctional chain extenders, and short glass fibers (GFs). The rPET-GF composites were characterized in terms of their processing, thermal, thermomechanical, and mechanical properties. Epoxy-functional chain extender modification effectively increased the molecular weight and improved the processability, whereas GF reinforcement enhanced the tensile properties of both injection-molded and FDM-manufactured parts. A primary advantage of the rPET systems developed in this study is their delayed crystallization kinetics. These findings highlight the significant potential of the composites developed herein for extrusion-based additive manufacturing (MEX-AM), as delayed crystallization facilitates enhanced interfacial adhesion, lower volumetric shrinkage, and superior dimensional stability.

## 1. Introduction

Polyethylene terephthalate (PET) is one of the most widely used and versatile polymers. Owing to its low weight, high mechanical performance, favorable thermal behavior, and transparency, it is widely used in packaging, textiles, and engineering applications [1,2]. The best-known commercial polyester product is the PET bottle, and its market is forecasted to exceed 68 billion dollars by 2034, reflecting an approximate 4% compound annual growth rate between 2024 and 2034 [3]. Parallel to economic growth, PET is also a central focus in sustainability research, as it is among the most widely recycled polymers globally. With a theoretical 100% recyclability, PET can be recycled through mechanical, chemical, and so-called bio-recycling (enzymatic) routes [2,4,5,6]. However, the current management of this material reveals a significant gap; according to recent estimates, only about 41% of the material reaches recycling or incineration facilities, while the rest is disposed of in landfills or released into the natural environment [2]. To address these inefficiencies, governments worldwide are implementing new legislative requirements and standards that are currently driving significant transformations in the global PET market [5,7,8,9]. In particular, deposit-return systems can improve the availability of recycled PET feedstock, helping reduce waste and increase the share of recycled materials in the market. For example, under the new European Union Packaging and Packaging Waste Regulation (PPWR), producers are required to gradually increase the share of recycled content; for PET packaging, the target is at least 30% by 2030 and 50% by 2040 [7]. To better align with the global circular economy objectives, China recently introduced a new set of guidelines for PET packaging. The standard, Plastics—Guide of Design for Recycling—Part 1: Poly(ethylene terephthalate) (PET) Materials (GB/T 46020.1-2025), entered into force in February 2026. Although currently non-mandatory, it serves as the primary national reference for the design of recyclable PET [5].

The mechanical recycling of PET remains the leading industrial technology. It accounts for approximately 73% of the recycled PET content [4]. This multistage approach, comprising collection, sorting, washing, separation, and extrusion, facilitates the recovery of polyester without the need for costly depolymerization or harmful solvents, thereby reducing production costs and minimizing the risk of secondary pollution. However, PET, like other condensation polyesters, is susceptible to thermal degradation, particularly via hydrolysis and alcoholysis at high processing temperatures [4,10]. Furthermore, in extrusion-based mechanical recycling, high shear forces in the melt promote shear-induced chain scission [10,11]. Both thermal and thermomechanical degradation reactions lower the molecular weight and adversely affect the rheological behavior and processability. The degradation of PET has been leveraged in the recycling of polyester bottles into fibers, where a lower melt viscosity facilitates melt spinning [12]; however, to adapt to additional technical applications, the polymer must be upgraded. The property deterioration of recycled PET can be compensated by blending [13], adding chemical chain extenders [12,14,15,16], and reinforcing fillers [17,18,19]. Furthermore, these strategies can be combined to achieve synergistic enhancement and produce engineering-grade rPET [20,21,22,23,24]. Recycled PET is extensively applied in the packaging, textiles, and engineering sectors, contributing to cost reduction and a lower carbon footprint [4]. However, to achieve maximum material recirculation, practical steps are required, including increasing recycling capacity and developing more robust markets for recycled materials. In this context, recent investigations have evaluated the possibility of using recycled PET (rPET) waste in additive manufacturing (AM), specifically focusing on material extrusion-based 3D printing technologies (MEX-AM) [12,25,26,27,28,29]. This strategy yields a twofold advantage: it decreases the ecological footprint of polymer production and provides a cost-effective feedstock for rapid prototyping.

In a related study, Rashwan et al. [25] reported that rPET pellets compounded with a chain extender, toughening agent, and impact modifiers could be successfully extruded into filament for MEX-AM technology. Four additives were utilized: (i) pyromellitic dianhydride (PMDA) as a chain extender, (ii) styrene–ethylene–butylene–styrene terpolymer functionalized with maleic anhydride (SEBS-g-MA) as a thermal modifier and toughening agent, (iii) ethylene–ethyl acrylate–glycidyl methacrylate terpolymer (E-EA-GMA) as a functional reactive elastomeric impact modifier, and (iv) ethylene-ethyl acrylate (EEA) as a non-reactive elastomeric impact modifier. The modified formulations exhibited superior melt strengths compared to neat rPET. Although certain defects were present in the 3D-printed samples, the modification resulted in notable enhancements in the mechanical performance, particularly in terms of stiffness, toughness, and structural integrity. Another study conducted by Hu et al. [12] demonstrated an innovative value-added approach for recycling polyester textiles into 3D printable filaments by modifying recycled polyester with an epoxy chain extender (Joncryl ADR4468). In their study, Vishal Mishra et al. [26] investigated the printability of various grades of PET, specifically virgin PET pellets (VPET), recycled PET pellets (RPET-P), and recycled PET bottle flakes (RPET-CF). This study systematically characterizes the temperature-dependent physio-mechanical behavior of three material grades, specifically focusing on the effects of processing at 250, 260, and 270 °C. This investigation identifies additive-free recycled PET (rPET), specifically in flake form, as a viable candidate for structural additive manufacturing applications. The authors claimed that increasing the processing temperature to 270 °C improved the interlayer adhesion, crystallinity, flexural rigidity, and tensile strength of RPET-CF. Research carried out by Andrzejewski et al. [27] investigated recycled PET-based blends containing two amorphous copolyesters: poly(ethylene terephthalate) glycol (PETG) and poly(cyclohexylenedimethylene terephthalate) glycol (PCTG). The 50/50 wt% rPET/copolymer compositions, compatibilized with a SAN-co-GMA chain extender, maintained excellent processability for FDM applications. Specifically, these formulations exhibited minimal shrinkage and no warping when 3D printed. The mechanical and thermal properties of these materials remained robust, with the potential for further enhancement through post-processing annealing. In a follow-up study, Andrzejewski et al. [29] examined composites based on the previously mentioned rPET/PCTG blend, along with fibrous CF and platelet talc as modifiers. The incorporation of fillers into the prepared system confirms the beneficial impact of fibrous reinforcement on the mechanical properties. Recently, complementary research conducted by Trossaert et al. [30] assessed the recyclability of PETG and PET in MEX-AM applications by incorporating industrial waste PETG and post-consumer PET waste into virgin PETG. The authors claimed that small amounts of post-industrial waste can enhance the mechanical properties. Conversely, post-consumer PET waste exhibited inconsistent, grade-dependent effects on the mechanical performance of PETG 3D-printed parts. Although the mechanical performance generally decreased with the addition of post-consumer waste, the 40% post-consumer PET content substantially improved the flexural strength of high-viscosity PETG printed parts (HV-grade). According to the authors, this resulted from the lubricating effect of degraded PET chains, which improved the flow, interlayer adhesion, and elongation in HV-grade PETG.

Another approach described in the literature is the direct extrusion of PET filaments or PET flakes. Nikam et al. [31] report that strips from waste PET bottles can be directly extruded into filament for AM. Although promising, this approach is difficult to scale up for industrial applications. While standard extrusion lines can seamlessly process conventional pellets or PET flakes, this technique requires an additional preliminary step: cutting waste bottles into strips. A slightly different approach was adopted by Little et al. [32], who investigated the potential for direct printing; specifically, they employed a method similar to direct pellet extrusion (DPE) utilizing washed PET flakes as the primary feedstock. The authors emphasized that implementing rigorous material-handling and drying protocols makes it possible to process flakes directly, avoiding the thermal and hydrolytic degradation that normally occurs during repeated processing.

Various recycled PET (rPET) compositions have been studied to date; however, to the best of our knowledge, none of the existing literature addresses glass fiber-reinforced rPET composites for FDM manufacturing. Simultaneously, it is widely recognized that the performance of 3D-printed parts can be greatly improved by incorporating carbon (CF) or glass fiber reinforcement (GF), a strategy that is especially important for applications requiring high structural stiffness and fracture toughness [33,34,35]. Moreover, while boosting the mechanical properties, both additives successfully reduced thermal deformation and enhanced processability by minimizing warping and providing excellent dimensional stability [36,37]. Considering the aforementioned, developing a scalable protocol for the mass production of AM-friendly, high-performance composites incorporating a high percentage of recycled PET waste remains an ongoing challenge. Given the importance of waste valorization and the increasing need for high-performance additive manufacturing materials, this study focused on rPET composites. The formulations developed herein employ an epoxy-functionalized chain extender (Jocryl^®^) to minimize molecular weight loss and short glass fibers as a cost-effective reinforcement that enhances mechanical performance. rPET composites with a constant chain extender content (1.5 wt%) and glass fiber loadings adjusted in the range of 5–15 wt% were investigated. The influence of the applied modification on the basic physicochemical, processing, thermal, and thermomechanical properties was evaluated. To better understand the mechanisms and kinetics of crystallization, non-isothermal crystallization kinetics were studied using DSC analysis. Finally, both injection-molded and 3D-printed specimens were subjected to mechanical characterization, and their tensile, flexural, and impact properties were examined in detail. The fractured surfaces of the impact test samples were also examined.

## 2. Materials and Methods

### 2.1. Materials

Post-consumer PET flakes (washed and shredded mixed-color soft drink bottles) were supplied by Ergis-Recycling Sp. z o.o., Biała, Poland. A general-purpose PETG copolyester (Selenis Selekt BD 110, Selenis, Portalegre, Portugal) was used to prepare the chain extender masterbatch. Short glass fibers (GFs), specifically milled E-glass fibers (FG 160/600) with a nominal fiber length of ~160 µm, fiber diameter of ~10 µm, featuring silane-based sizing for polyester compatibility supplied by BASF (Ludwigshafen, Germany), were used as filler. The detailed characteristics of the filler are provided elsewhere [38]. The functional additive epoxy-functional styrene–acrylic oligomeric chain extender Joncryl ADR4368 was obtained from BASF Corporation, Ludwigshafen, Germany. According to the manufacturer’s specifications, Joncryl ADR4368 has a specific gravity of 1.08 g/cm^3^ (25 °C), a weight-average molecular weight (M_w_) of 6800 g/mol, a glass transition temperature of 54 °C, and an epoxy equivalent weight of 285 g/eq.

All polymeric materials were dried in-line in a vacuum oven (Binder VD23, Tuttlingen, Germany) at 110 °C (PET flakes) or 60 °C (PETG and PETG-based Joncryl masterbatch) and 7 mbar for 12 h prior to processing. The GFs were dried for approximately 12 h at 100 °C in a convection oven (POL-EKO, Kędzierzyn-Koźle, Poland). The components were fed using Brabender gravimetric feeders (Brabender, Duisburg, Germany). Two dosing methodologies were tested for the chain extender: one-step direct feeding and two-step masterbatch extrusion (Joncryl on a PETG carrier) followed by PET flake compounding. In the direct approach, PET flakes were compounded with 1.5 wt% Joncryl ADR using a lab-scale counter-rotating twin-screw extruder (Laborextruder LSM30 L/D = 22.9, D = 34 mm, Leistriz, Nürnberg, Germany), maintaining a temperature profile from 160 to 260 °C (from feed to nozzle). The two-stage process featured the production of Joncryl masterbatches using a minimized temperature profile to prevent chain extender activation (feeding zone set at 160 °C, followed by zones with temperatures set to 220 °C). A masterbatch incorporating 5 wt% chain extender was obtained. In the subsequent step, the granulated and dried product was combined with PET flakes in a 70:30 weight ratio (PET flakes:masterbatch) to yield blends with 1.5 wt% chain extender concentration. Components were fed to the extruder throat via a dual-feed gravimetric system (Brabender, Duisburg, Germany). For the unfilled rPET formulation, the Joncryl masterbatch and PET flakes were metered using separate feeders. For the composites, the setup was modified such that the chain extender masterbatch was fed through the first feeder, whereas a premix of PET flakes and glass fibers was introduced via the second feeder. Three formulations were prepared with a target chain extender content of 1.5 wt% and GF loadings of 5, 10, and 15 wt%. The final compositions were processed at temperatures ranging from 190 to 260 °C. With the feeder output set to 1.8 kg/h and a screw speed of 50 RPM, it took ~3 min for the material to pass from the feed section to the extruder nozzle. The extruded filaments were quenched in a water bath and pelletized.

The obtained composites and a chain-extended rPET reference material exhibited adequate melt strength for injection molding and additive manufacturing applications. For tensile testing, ISO 527-2 5A [39] dog-bone specimens (gauge length: 20 mm, gauge width: 4 mm, thickness: 2 mm) and ISO 180 [40] Izod impact bars (80 mm × 10 mm × 4 mm) were prepared using a Fanuc Roboshot α-100iB injection-molding machine (FANUC, Oshino, Yamanashi, Japan) characterised by a 1000 kN clamping force and 26 mm screw diameter. For 3D printing tests, the materials were extruded into calibrated filaments (1.75 ± 0.05 mm diameter) using a Filament Maker ONE Precision 450 single-screw extruder (3devo, Utrecht, The Netherlands). Tensile and impact test specimens, sharing the same nominal geometry as the injection-molded samples, were printed using a Bambu Lab X1 Carbon FDM printer (Shenzhen, China) with a 0.4 mm nozzle. The 3D-printed samples for the Izod impact evaluation were manufactured with full density (100% infill) and 45° raster angle, without any stress concentrators (unnotched). The same printing conditions were used for all the investigated materials. The typical processing parameters are listed in Table 1.

**Figure 1:**
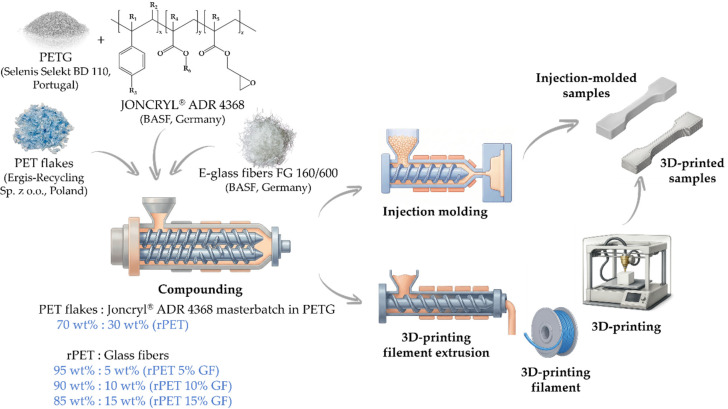
The materials and processing pathways employed in the production and processing of rPET and its composites.

A schematic representation of the workflow is shown in Figure 1.

### 2.2. Characterization Methods

Illustrations in the graphical abstract and some parts of Figure 1 were generated using an AI-powered scientific illustration platform, Illustrae (Version 2026).

The intrinsic viscosity (IV) of the samples was measured using a capillary Ubbelohde (type 1c, K = 0.03294; Labit, Koczargi Nowe, Poland) at 30.0 ± 0.1 °C. Polymer solutions (5 g/dL) were prepared in phenol/1,1,2,2-tetrachloroethane (60:40 by weight) purchased from Sigma-Aldrich (St. Louis, MO, USA). To eliminate the influence of the GF, composite samples were dissolved in a mixture of phenol/1,1,2,2-tetrachloroethane (60:40 by weight) and filtered through a membrane filter with a 0.8 μm pore size. The samples were then precipitated with methanol. The obtained precipitate was dried under vacuum at 60 °C for 12 h and redissolved.

Density measurements were performed at 23 °C using a hydrostatic balance (Radwag AS160 C2, Radom, Poland) with distilled water as the immersion medium.

Loss on drying (LoD) was quantified using a thermogravimetric method based on the weight loss of the granules during controlled heating. The measurements were performed using a Radwag MAX 60/NP halogen moisture analyzer (Radwag, Radom, Poland) on samples of approximately 10 g at 80 °C. The process ended when the sample mass stabilized within 1 mg for a predetermined duration, confirming complete dehydration.

Melt flow rate (MFR) measurements were performed using a CEAST 6841 flow tester (CEAST, Turin, Italy), according to ISO 1133 [41]. Tests were conducted at 285 °C under a 2.16 kg load, using a standard die (diameter: 2.095 mm; length: 8.000 mm). All materials were vacuum-dried at 100 °C prior to testing.

The characteristic phase transition temperatures, including the glass transition (*T_g_*), melting (*T_m_*), and crystallization (*T_c_*) temperatures, were analyzed using a DSC 204 F1 Phoenix^®^ calorimeter (Netzsch, Selb, Germany). Each DSC testing cycle consisted of heating–cooling and repeating the scans, with a heating/cooling rate of 10 °C/min, from 0 to 250 °C. The results of the second heating run were used for the investigation. The heat of fusion (Δ*H_m_*) and crystallization (Δ*H_c_*) were calculated from the total areas under the melting and crystallization peaks on the DSC curve. The degree of crystallinity (*x_c_*) was calculated from the enthalpy of melting according to Equation (1):

(1) $$x_{c}=\left(\frac{\Delta H_{m}}{w_{PET}\cdot \Delta H_{m}^{0}}\;\right)\cdot 100\;\left[\%\right]$$

where Δ*H_m_*—melting enthalpy [J/g], $\Delta H_{m}^{0}$—theoretical melting enthalpy value for 100% crystalline PET ($\Delta H_{m}^{0}$ = 144.5 J/g), $w_{PET}$—weight fraction of *PET*.

Non-isothermal crystallization was studied using a multi-cycle heating/cooling program using a DSC 204 F1 Phoenix^®^ calorimeter (Netzsch, Selb, Germany). The sample was heated from 0 to 270 °C at a rate of 10 °C/min, followed by a 5 min annealing step at 270 °C to erase the sample’s thermal history. Subsequent cooling to 0 °C was performed at rates of 10, 7.5, 5, and 2.5 °C/min. Kinetic data were derived from cooling scans.

The viscoelastic properties of rPET and its composites were analyzed using a dynamic thermomechanical analyzer (DMA 303 Eplexor, Netzsch, Selb, Germany). The storage modulus (E′) and loss modulus (E″) were measured as a function of temperature using the dual-cantilever mode at a fixed frequency of 1 Hz from −70 to 200 °C with a heating rate of 3 °C/min. ISO 527 5A dog-bone specimens, with a gauge length of 20 mm, a gauge width of 4 mm, and a thickness of 2 mm, were used for the tests. The DTMA results are expressed as the storage modulus (E′) corresponding to the elastic response to deformation and the mechanical loss factor (tan δ) versus temperature.

The mechanical properties under static tension were recorded using a universal testing machine Autograph AG-X plus (Shimadzu, Kyoto, Japan) equipped with a 1 kN load cell, a non-contact TrViewX video extensometer, and pneumatic side clamp grips. The measurements were carried out in accordance with the PN-ISO 527 standard at a crosshead speed of 1 mm/min up to 1% elongation, followed by 5 mm/min to break. The tests were conducted at 23 °C and 50 ± 5% relative humidity. Key properties, including tensile modulus (Young’s), strength and elongation at yield (σ_y_, ε_y_), and strength and elongation at break (σ_b_, ε_b_), were determined from at least seven measurements per material and are presented as representative stress–strain curves and mean ± standard deviation values.

The impact strength was tested using the Izod method according to the EN ISO 180 standard on the pendulum impact tester B5102 manufactured by Zwick/Roell, Ulm, Germany, with a pendulum capacity of 2 J. Notch type A (2 mm depth, 45° angle, 0.25 mm radius of notch base) was machined using a dedicated notching machine. The tests were conducted at 23 °C and 50 ± 5% relative humidity. The reported values are the averages of ten samples.

The fracture surfaces close to the notch regions of the representative samples were analyzed using a digital microscope 4 K VHX-7000 (Keyence, Osaka, Japan) with a 30× optical zoom.

## 3. Results and Discussion

### 3.1. Primary Characterization of Chain-Extended rPET and Its Composites

Chain-extended rPET and composites thereof with glass fiber loadings of 5, 10, and 15 wt% were manufactured and analyzed in detail. Epoxy-functional Joncryl 4368 was used as a chain extender. Screening studies using different modifier concentrations and dosing strategies have been performed. It has been found that stable extrusion could not be achieved without or with a lower amount of a chain extender (low melt strength, polymer dripping from the nozzle) or via direct chain extender dosing (an unstable process). The issues regarding the stable processing of neat rPET align with the observations of Restrepo et al. [42] and Andrzejewski et al. [27]. In the present study, optimal results were obtained using a Joncryl–PETG masterbatch (30 wt% premix with 5 wt% Joncryl content, corresponding to 1.5 wt% overall extender concentration in the composition). Recent studies [24] have validated a comparable two-stage incorporation method for chain extenders. The latter has proven to be highly effective in reconstructing the molecular architecture of rPET and restoring its physical properties. The basic physical and processing properties are summarized in Table 2. The IV of the chain-extended rPET reached a maximum value of 0.97 dL/g, whereas the values for the composites were lower by 0.03–0.04 dL/g. The authors attributed this effect to the fact that the presence of the GF rendered the melt far more viscous, making it difficult for the chain extender to diffuse out during the reactive extrusion process. Consequently, the chain extension reaction was retarded, resulting in a reduced intrinsic viscosity (IV) and, hence, a lower molecular weight of the rPET composites at identical modifier concentrations. Nonetheless, the IV of all recycled formulations was sufficiently high for injection-molding and extrusion-processing applications. The chain-extending effect of Joncryl ADR is also confirmed by the relatively high melt strength, which manifests as a low melt flow rate value (MFR_285 °C, 2.16 kg_ = 10.7 g/10 min). Wu W.J. et al. [43] reported comparable results using 1.5 wt% Joncryl chain extender with similar functionality for PET fiber recycling, yielding an IV of 0.86 dL/g and MFR of 13.4 g/10 min. In our study, a further decrease in MFR was observed with increasing GF loading, likely resulting from restricted polymer chain mobility coupled with increased fiber–fiber and fiber–matrix friction. The lowest MFR value of 3.4 g/10 min was achieved in the rPET 15% GF composite.

The incorporation of high-density silica glass fibers (d = 2.63 g/cm^3^ [38]) into the rPET matrix increased the overall density of the composites. Following the rule of mixtures, the measured density increased from 1.31 g/cm^3^ for the unreinforced rPET to 1.41 g/cm^3^ at a 15 wt% GF loading.

**Figure 2:**
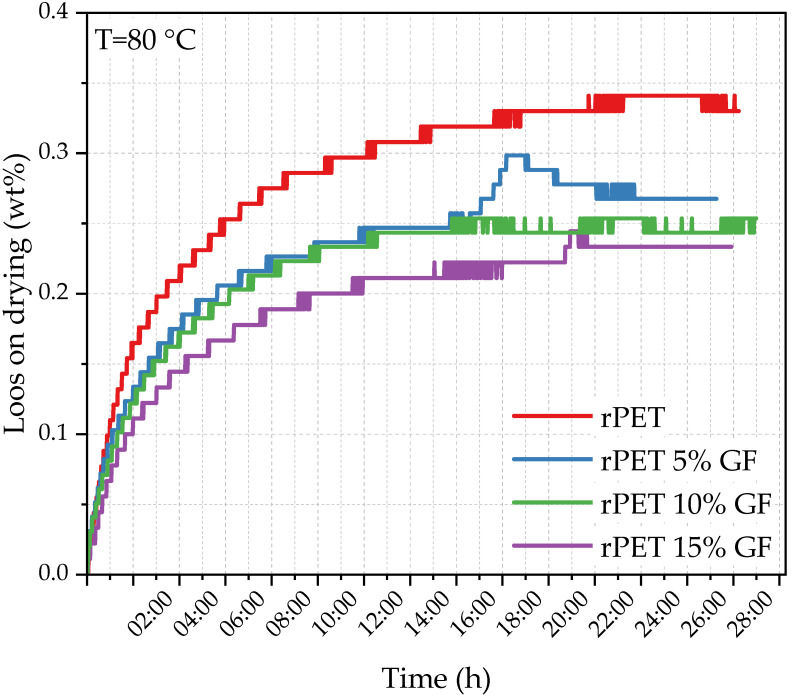
Loss on drying as a function of time for rPET and glass fiber composites.

It is well known that polyesters with backbones incorporating heteroatoms are particularly prone to hydrolytic degradation [44,45]. Therefore, maintaining the moisture content at minimal levels across all stages of processing is essential to achieve the optimal properties of the material. It is well established that chain extenders can minimize water uptake by increasing the molecular mass and chain entanglement [14]. The weight loss profiles during drying at 80 °C are illustrated in Figure 2. All samples were kept at room temperature (45% relative humidity) for two weeks prior to measurement. With a fixed chain extender content, the loss on drying (LoD) decreases from 0.34 to 0.23 wt% as the GF loading increases from 0 to 15 wt% (Table 2). It is likely that the lower hydrophilic polymer content, combined with fibers creating tortuous diffusion paths, accounts for this observation. This is in agreement with the findings of Tolcha et al. [46], who observed a pronounced decrease in the water uptake of glass fiber-reinforced PP/PET composites compared with that of unfilled systems.

### 3.2. Thermal Properties and Crystallization Kinetics

**Figure 3:**
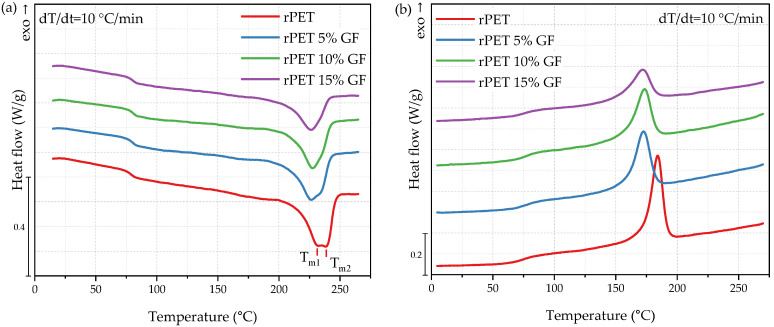
DSC thermograms recorded during (**a**) second heating and (**b**) cooling of chain-extended rPET and its glass fiber composites.

The DSC scans of the investigated materials are shown in Figure 3, and the important thermal transition data and crystallinity degrees are summarized in Table 3. All samples exhibited the typical profile of semi-crystalline materials, with a glass-to-rubber transition at lower temperatures, followed by an endothermic phenomenon associated with the melting of the crystalline phase in the high-temperature region. Virgin PET tested under the same conditions exhibits a slightly higher glass transition temperature (85.5 vs. 78.3 °C), as well as increased crystallization (203.6 vs. 185.0 °C) and melting temperatures (248.4 vs. 232.1/238.4 °C), accompanied by a higher degree of crystallinity (30.1 vs. 16.5%) [47]. The observation of a single *T_g_* in the rPET/PETG (Joncryl masterbatch) system was already expected. This is due to the miscibility of the two polyesters [27], which is further enhanced by the chain extender, which acts as a compatibilizer [14]. Although the GF modification did not significantly shift *T_g_*, the values were slightly higher for the composites, increasing from 78.3 °C for the neat rPET to 79.6–80.4 °C for the GF composites. The corresponding changes in the heat capacity increment (Δ*C_p_*) remain comparable across the series (0.15–0.17 J/g*K).

Among the studied systems, rPET exhibited the highest melting temperatures, characterized by a double peak at 232.1 and 238.4 °C. This behavior is common in PET and typically originates from either the existence of multiple crystalline phases or a melting–recrystallization–melting phenomenon [48,49]. For the rPET/GF composites, differences in the melting and crystallization peak positions, multiplicity, and peak intensity were observed. The crystallizability of rPET was clearly reduced with increasing GF loading, as evidenced by decreases in *T_m_* (from 232.1/238.4 to 225.5 °C) and Δ*H_m_* (from 32.9 to 19.7 J/g). Along with lower peak melting temperatures, lower crystallization temperatures and crystallization enthalpies were also observed; *T_c_* decreased from 185.0 °C to 172.8 °C, and Δ*H_c_* decreased from 32.8 to 19.7 J/g (see Table 3). This thermal response implies that the composites cannot crystallize as fast as rPET, which eventually results in a higher amount of the amorphous phase after cooling from the melt. Indeed, *x_c_* dropped from 16.5% for neat rPET to 13.4% at a 5 wt% GF loading (approximately 80% of the neat polymer’s value) and continued to decline, reaching 9.9% at a 15 wt% GF loading (retaining only 60% of the neat rPET value). Based on the Δ*H_m_* values obtained from the first heating curves (Supplementary Information, Figure S1 and Table S1), a clear trend, specifically a reduction in crystalline-phase content as GF loading increases, is evident for both the injection molded and 3D-printed samples. Furthermore, the specific melting enthalpies of the injection-molded specimens are significantly lower than those of the corresponding 3D-printed samples, indicating a pronounced effect of the processing route on the material’s crystalline structure. The literature reports contradictory results regarding the impact of both the chain extender and GF filler on polymer crystallinity. From one perspective, chain extension may suppress the formation of lamellae, as the higher molecular weight reduces polymer chain mobility [50]. The formation of a partially crosslinked structure with several branching points that act as nucleation centers was previously reported by Wang et al. [51] and Andrzejewski et al. [52] for PLA, another member of the polyester family. The influence of glass fibers on the crystallinity of PET/GF composites also remains debated. Certain authors claimed that adding GF promotes PET crystallization [53], while others suggested a reduction in PET crystallinity upon GF incorporation [54]. A third view suggests a concentration-dependent effect, in which low GF loadings serve as nucleating agents, whereas higher loadings physically impede the crystal growth [55]. Herein, the effects of the chain extender and GF are difficult to separate; nonetheless, it is evident that GF incorporation alters the kinetics and thermodynamics of crystallization from the melt. These observations are crucial for advancing fundamental knowledge and practical applications, particularly in technological frameworks focused on Fused Deposition Modeling (FDM) additive manufacturing. Optimal materials in this method feature low processing temperatures, an amorphous character, or retarded crystallization, with crystallization time remaining the primary factor governing the printability of advanced semi-crystalline polymers [28,56,57]. To shed light on this, non-isothermal crystallization experiments were conducted on rPET and its GF-reinforced composites.

**Figure 4:**
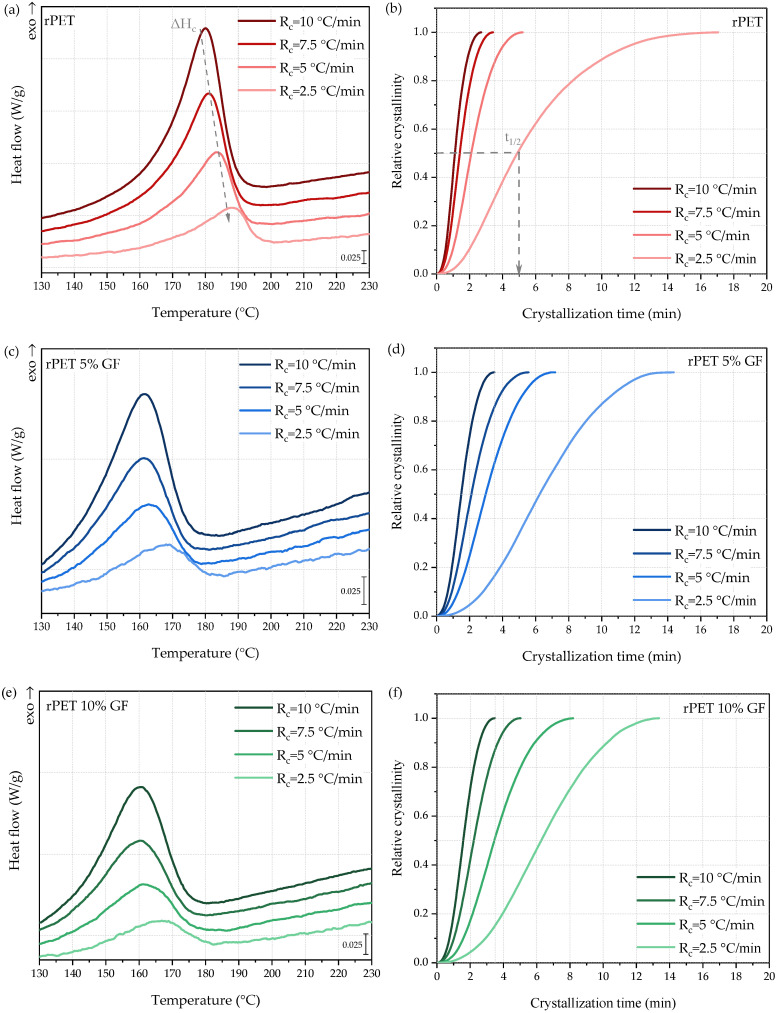

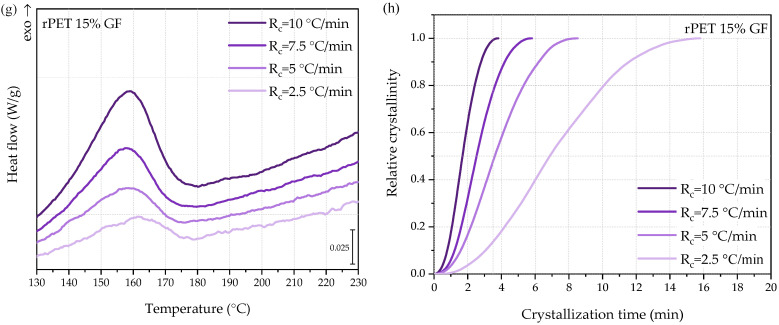
Crystallization exotherms for (**a**) rPET, (**c**) rPET 5% GF, (**e**) rPET 10% GF and (**g**) rPET 15% GF, along with the corresponding relative degree of crystallinity vs. time for (**b**) rPET, (**d**) rPET 5% GF, (**f**) rPET 10% GF and (**h**) rPET 15% GF at different cooling rates.

The non-isothermal crystallization of the rPET and rPET/GF composites was evaluated by DSC using cooling rates of 2.5, 5, 7.5, and 10 °C/min. Figure 4 shows the crystallization exotherms (left panel) and the evolution of the relative degree of crystallinity over time (right panel). The relative degree of crystallinity, X(T), was determined by integrating the crystallization exotherms according to Equation (2).

(2) $$X\left(T\right)=\frac{\int_{T_{0}}^{T}(dH_{c}/dT)dT}{\int_{T_{0}}^{T_{\infty }}(dH_{c}/dT)dT}$$

where *T*_0_ and *T*_∞_—temperatures at the start and end of crystallization process, respectively; *dH_c_*/*dT*—heat flow rate.

Subsequently, the relative crystallinity as a function of time, *X*(*t*), was evaluated as follows: The latter was calculated using the straightforward relationship between crystallization time (*t*), temperature, and cooling rate, as described by Equation (3).

(3) $$t=\frac{T_{0}-T}{R_{c}}\;$$

where *T*_0_—temperature at the start of crystallization; *T*—temperature at time *t*; *R_c_*—cooling rate.

The corresponding thermal parameters, together with crystallization half-time (t_1/2_), are detailed in Table 4. Figure 4 (left panel) reveals a strong and well-documented correlation between R_c_ and the thermal phenomena associated with crystallization [58,59,60]. In all the studied systems, increasing R_c_ broadened the phase transition. Higher cooling rates also systematically shifted the onset (*T*_0_) and peak (*T_c_*) crystallization temperatures to lower values, while simultaneously reducing the crystallization enthalpy (Δ*H_c_*). In other words, fast cooling delays polymer chain organization and induces a more defective crystalline phase driven by the formation of thinner and less perfect lamellae. As far as the reinforcement influence is considered, the steady decline in *T*_0_, *T_c_*, and Δ*H_c_* values across all cooling rates demonstrates the significant effect of GF on the crystallization behavior of rPET. The evolution of the relative degree of crystallinity (*X*(*t*)) for rPET and its composites cooled from the melt at various rates is illustrated in Figure 4 (right panel). The *X*(*t*) profiles exhibited a characteristic sigmoidal shape, representing the distinct stages of the crystallization process, that is, nucleation, nuclei growth, and spherulite impingement [58]. The crystallization half-time (t_1/2_), that is, the time required to reach 50% relative crystallinity at a specific cooling rate, was determined (Table 4). As can be seen, the t_1/2_ typically decreases as the cooling rate increases. For neat rPET, t_1/2_ decreased from 5.02 min to 1.12 min as the cooling rate increased from 2.5 °C/min to 10 °C/min. Furthermore, a comparison of the t_1/2_ data at identical cooling rates clearly demonstrates that even minor GF loadings significantly increase the crystallization half-time. The t_1/2_ values recorded for the rPET/GF composites exceeded 6 min, reaching nearly 7 min for the 15% GF loading at R_c_ = 2.5 °C/min. These findings are especially significant, as the observed values align closely with the crystallization half-times characteristic of PLA, a polymer known for its slow crystallization rate and one of the most frequently used semi-crystalline polymers in additive manufacturing [61]. The latter does not crystallize at a cooling rate of 10 °C/min and exhibits a t_1/2_ of 6 min when cooled at 2 °C/min [59]. In the context of 3D printing, specifically FDM, a reduction in the crystallinity and crystallization rate is highly favorable. In an ideal scenario, subsequent layers in 3D printing should be deposited onto a substrate in an amorphous state to ensure sufficient bonding. The primary difficulty involves deposition onto highly crystallized polymer layers, which act as a thermal barrier that restricts macromolecular diffusion at the interface, consequently leading to interlaminar defects and *Z*-axis anisotropy of the composite. Furthermore, crystal lattice formation during and after layer deposition induces volumetric shrinkage and residual internal stresses, often resulting in insufficient bed adhesion, low dimensional stability, and warping [62,63]. Although 3D printing with fast-crystallizing thermoplastics such as polyamide 6 (PA6), polyether ether ketone (PEEK), and polypropylene (PP) is technically feasible, processing challenges often limit their broader application. In contrast to the nearly instantaneous crystallization observed in PA6 (t_1/2_ < 1 min at dT/dt = 10 °C/min) [60], PEEK (t_1/2_ ~ 0.49 min at dT/dt = 10 °C/min) [64], or PP (t_1/2_ ~ 0.53 min at dT/dt = 10 °C/min) [58], the rPET composites characterized herein demonstrate significantly slower crystallization rates (t_1/2_ of 1.52–1.71 min at dT/dt = 10 °C/min, depending on GF loading).

**Figure 5:**
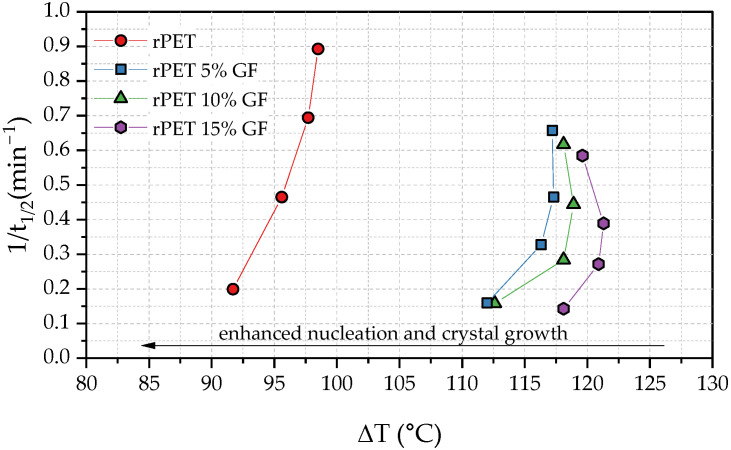
Dependence of 1/t_1/2_ on supercooling for rPET and rPET/GF composites with varying glass fiber loadings.

In order to effectively correlate compositional changes with crystallization phenomena, the relationship between 1/t_1/2_ and degree of supercooling (Δ*T*) was plotted (Figure 5), where Δ*T* is defined as the difference between the equilibrium melting temperature, T_m_^0^ (280 °C for PET [65]), and the recorded crystallization temperature. It is evident that the incorporation of GF hinders the ordered packing of rPET chains; consequently, the crystallization rate is significantly reduced in the composites and continues to decrease as the GF loading increases. This trend was quantitatively supported by the lower 1/t_1/2_ values recorded for the composite samples. In particular, rPET with the highest GF loading requires a significantly higher degree of supercooling to initiate the non-isothermal crystallization process (22–27 °C more than neat rPET). This behavior is another strong suggestion that, in the investigated systems, the steric hindrance imposed by the GF outweighs any potential heterogeneous nucleation effect, thereby retarding the diffusion-controlled growth of rPET crystals [58].

Similar to low-molecular-weight substances, the kinetics of polymer crystallization under isothermal conditions are typically analyzed using Avrami’s classical theory [66]. In general, the Avrami model (Equations (4) and (5)) is most effective at describing the early stages of the crystallization process, assuming steady growth, first-order nucleation, and statistically distributed nuclei [59,60,67].

(4) $$1-X\left(t\right)=\exp\left(-Z_{t}t^{n}\right)$$

(5) $$\ln\left(-\ln\left(1-X\left(t\right)\right)\right)=lnZ_{t}+n\;ln(t)\;$$

where *X*(*t*) is the relative degree of crystallinity, *Z_t_* is the crystallization rate constant (min^−n^), *t* is the time (min), and *n* is the Avrami exponent depending on the nucleation mechanism and crystal growth mode.

The same equation applies to the non-isothermal crystallization process, although the effect of the cooling rate (*R_c_*) on the nucleation and crystallization processes cannot be neglected. To address this, Jeziorny [68] suggested that the kinetic rate constant during isothermal crystallization should be corrected as follows:

(6) $$lnZ_{c}=\frac{lnZ_{t}}{R_{c}}$$

**Figure 6:**
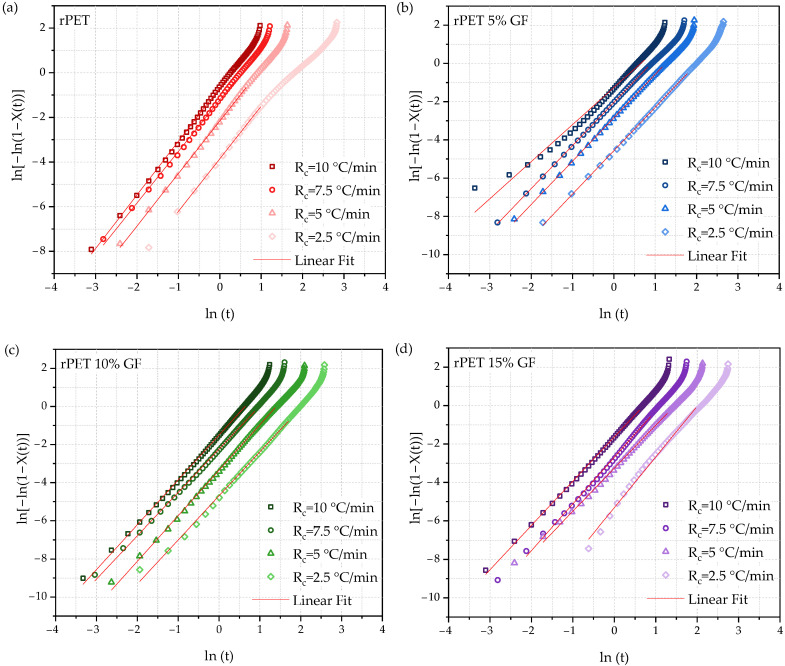
Avrami plots of ln[−ln(1 − *X*(*t*))] vs. ln(*t*) for non-isothermal crystallization of (**a**) rPET and rPET/GF composites: (**b**) 5% GF, (**c**) 10% GF, and (**d**) 15% GF.

The plots of ln[−ln(1 − *X*(*t*)) vs. ln(*t*) for rPET and rPET/GF composites are shown in Figure 6. By fitting a linear regression (red lines) to the initial linear regions of these curves, the Avrami exponent (*n*) and crystallization rate constant (*Z_t_*) were extracted from the slope and intercept, respectively. These parameters, including the calculated Jeziorny-modified constants (*Z_c_*), are listed in Table 4. Given that the *R*^2^ values remained above 0.998 for all samples, it can be concluded that the linear region of the Avrami plot accurately represents the primary crystallization behavior of rPET. The obtained results facilitate a more comprehensive analysis of the nucleation mechanism, crystal growth mode, and crystallization rate. For primary rPET crystallization, the Avrami exponent was found to vary between 2.36 and 2.40 at a given cooling rate. Similar results were reported for PET by Jeziorny (*n* = 2.35–2.65 for cooling at 8.5–17 °C/min) and were attributed to three-dimensional spherulite growth and heterogeneous nucleation [68]. Notably, in the presence of GF, the n value was considerably reduced to approximately 2 for all cooling rates, implying two-dimensional lamellar growth and heterogeneous nucleation [67]. In fact, since the Avrami exponents are non-integers, the crystallization mechanisms of the rPET and rPET/GF composites must be regarded as mixed. Three-dimensional spherulitic growth was dominant in rPET, whereas two-dimensional lamellar growth dominated in the rPET/GF composites. Therefore, short glass fibers act as physical obstacles, limiting the space for crystal development and hindering the transport of rPET chains in the interspherulitic regions, ultimately restricting the spatial dimensions of crystal growth.

For a fixed GF content, increasing the cooling rate slightly increased the *Z_t_* and *Z_c_* values, suggesting a higher crystallization rate. At the same cooling rate, increasing the GF loading decreased both *Z_t_* and *Z_c_*. This confirms that higher concentrations of glass fibers hindered the non-isothermal crystallization of rPET. This observation further corroborates the conclusions drawn from the t_1/2_ data. The role of molecular weight in crystallization should not be overlooked. The investigated rPET and its composites exhibited a high molecular weight (IV values of 0.93–0.97 dL/g), which can make the transport of chain segments to the crystal growth front more difficult, particularly for longer chains affected by entanglement, ultimately contributing to a low crystallization rate [1].

### 3.3. Thermomechanical Properties

**Figure 7:**
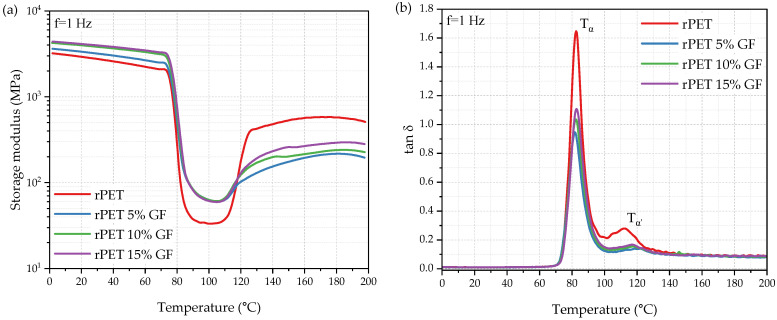
DMTA thermograms of chain-extended rPET and rPET/glass fiber composites. Plots of (**a**) storage modulus and (**b**) tan δ vs. temperature.

Dynamic thermal mechanical analysis (DMTA) was performed for further characterization. The thermograms of the storage modulus and tanδ of the rPET and rPET/GF composites are shown in Figure 7, and the relevant data are listed in Table 5. Between 0 °C and 70 °C, the E′ of all tested materials remained constant and relatively high, which is characteristic of the glassy state. The incorporation of GF significantly influenced the storage modulus within this temperature range. As summarized in Table 4, at room temperature, the E′ values of the 5%, 10%, and 15% glass fiber-reinforced composites reached 3300, 3900, and 4000 MPa, respectively, demonstrating a clear enhancement over the 2800 MPa observed for neat rPET. This was attributed to the restriction effect of the GF dispersed within the polymeric matrix. Short glass fibers restrict chain mobility and improve the overall stiffness of the composite.

As the temperature increased further, the polymer chains gained higher mobility and became more susceptible to deformation under the applied stress. E′ decreases significantly owing to the glass-to-rubber transition, and the energy dissipated by this increased segmental motion manifests as a sharp α-relaxation peak on the tanδ curve (dynamic representation of *T_g_*) (Figure 7b). The observed transition is consistent with the experimental data provided by Zahder et al. [69] for commercial-grade PET, rapidly cooled rPET filament and rPET samples produced via additive manufacturing. Forestier et al. observed comparable E′ and tanδ profiles for virgin PET in DMTA measurements, with a glass transition near 80 °C and a crystallization temperature of approximately 110 °C [70]. Although the tanδ peak position remained stable across all formulations (81.8–82.7 °C), its magnitude varied significantly, providing insight into chain dynamics. Compared with neat rPET, the composite materials exhibited restricted macromolecular mobility, as indicated by the decreased tanδ at T_α_. Despite this overall reduction, progressively increasing the glass fiber content from 5% to 15% actually improved macromolecular mobility, as evidenced by the increase in tanδ values at T_α_ from 0.9 to 1.1. The latter may also stem from enhanced stick–slip dissipation arising from the higher matrix-to-filler interface ratio, though these mechanisms are difficult to isolate. Once *T_g_* is exceeded, the polymer chains in the rPET and rPET/GF composites acquire sufficient mobility to fold and reorganize, with cold crystallization evidenced by the corresponding increase in E′ in the rubbery regime. While this effect is a recognized characteristic of PET and slow-crystallizing PET formulations, its intensity can vary substantially, largely depending on the origin of the polyester and recyclate composition [27,69]. Notably, the increase in the storage modulus in the rubbery region was more evident in rPET than in the composites. This suggests that the incorporation of the GF hinders the polymer’s ability to undergo not only melt crystallization (as evidenced in Section 3.2) but also cold crystallization. However, increasing the GF content slightly increased the stiffness within the elastic range. Two main factors account for this trend: (i) an increase in chain mobility (evidenced by a higher T_α_ peak intensity), which promotes cold crystallization, and (ii) the inherent rigidity introduced by higher GF loadings. The DSC thermograms recorded during the initial heating cycle for both injection molded and MEX-AM samples (Supplementary Information, Figure S1 and Table S1) also reveal exothermic cold crystallization peaks. This transition mirrors the crystallization behavior observed in the E′ curves, facilitating a cross-comparison between thermal and thermomechanical characterization of rPET and rPET/GF composites.

The second peak observed on the tan δ curve (T_α_′) reflects relaxation processes linked to cold crystallization. In addition to having a smaller magnitude, the T_α_′ relaxation peak is shifted 7–10° toward higher temperatures, rising from 110.7° for rPET to 120.3° for the 5% GF rPET composite. The corresponding increase in E′ supports this, confirming the growth of crystalline structures within the rubbery regime.

### 3.4. Mechanical Properties

**Figure 8:**
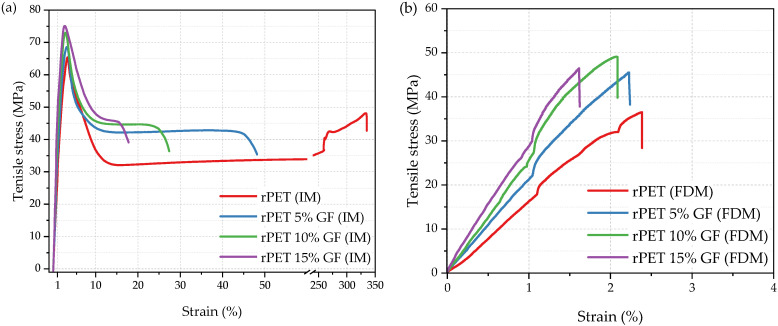
Representative tensile stress–strain curves of chain-extended rPET and rPET/glass fiber composites: specimens obtained via injection molding (**a**) and FDM 3D printing (**b**).

To gain some insight into the mechanical behavior, tensile, flexural, and impact properties were investigated for both injection-molded and FDM-manufactured samples, abbreviated further as IM and FDM, respectively. Representative stress–strain response curves are shown in Figure 8, and the important numerical values are summarized in Table 6. Preliminary inspection indicated that the manufacturing method was the dominant factor governing the tensile properties of the specimens. It is well established in the literature that the mechanical properties are significantly affected by the manufacturing method [27,71,72,73]. Compared to additive manufacturing, injection molding typically yields specimens characterized by superior and more isotropic mechanical properties. This performance advantage is primarily due to the enhanced material densification, negligible porosity, and superior structural homogeneity. In contrast, the mechanical behavior of MEX-AM components is largely controlled by the strength of the bonds between polymer strands and is strongly affected by the raster angle and deposition strategy [71]. Herein, injection molding yielded a more ductile material response (Figure 8a), in contrast to the inherent brittleness and rigidity of the additively manufactured specimens (Figure 8b).

Regarding the injection-molded specimens, the rPET exhibited a Young’s modulus (E_T_) of 3.1 GPa, a yield stress (σ_y_) of 65.4 MPa, and an elongation at break (ε_b_) of 333.8%. Benchmarking these results against the established literature values for unmodified virgin PET [49] shows that rPET is characterized by increased stiffness (E_T_ increase by 40%) and reduced ductility (ε_b_ decrease by approximately 33%). Importantly, σ_y_ remained essentially unchanged at approximately 65 MPa, indicating that the onset of plastic deformation in the rPET formulation was at the same level as that in the virgin polyester.

The addition of GF leads to higher tensile strength, stiffness, and rigidity, but at the cost of reduced ductility, as measured by the percentage elongation. Although the stress–strain curves of the IM composites show a distinct yield point, the ε_b_ for the 5 wt% GF sample dropped from 333.8% to 42.6%, that is, by approximately 87% relative to the rPET homopolymer. Simultaneously, σ_y_ increased from 65.4 MPa to 67.7 MPa. This effect became more pronounced with increasing GF content; the composites containing 10 and 15 wt% GF exhibited yield strengths of approximately 71.9 MPa and 75.1 MPa, respectively. With increasing GF weight fraction, the stiffness increased progressively, as Young’s modulus (E_T_) rose from 3.1 GPa for rPET to 4.4 GPa for rPET with 15% GF. The latter was accompanied by a progressive decrease in the elongation at yield (ε_y_) from 3.2 to 2.7%. The 3D-printed stress–strain curves (Figure 8b) display typical brittle behavior, with linear deformation up to fracture, without yielding and necking phenomena. The values obtained for rPET FDM, namely, E_T_ of 1.1 GPa, σ_b_ of 36.4 MPa, and ε_b_ of 2.5%, are consistent with those reported by Zander et al. [69] and Rashwan et al. [25] for unfilled 3D-printed recycled PET. The addition of GF substantially increased the σ_b_ of the composites relative to the unreinforced rPET matrix, with the 10 wt% GF sample achieving the highest value of 47.9 MPa. Concurrently, Young’s modulus E_T_ increased progressively from 1.2 to 3.0 GPa, while the elongation at break ε_b_ decreased from 2.5% to 1.7% with increasing GF content. Such behavior is typical of short fiber-reinforced thermoplastics [74,75], where stress concentrations from fibers and poor fiber–matrix adhesion usually trigger early crack formation. Moreover, the filler suppressed the plastic deformation of the matrix, promoting fracture rather than yielding. Notably, the mechanical performance of the FDM-printed samples was comparable to or superior to that reported for other rPET systems based on recycled bottles or textiles. Specifically, Rashwan et al. reported tensile strengths of around 31–32 MPa for neat rPET FDM specimens, increasing to approximately 38–42 MPa with impact modifiers; in comparison, our 10 wt% GF composite reached 47.9 MPa [25]. Similarly, Hu et al. reported tensile strengths of about 35 MPa for chain-extended rPET derived from recycled textiles [12], whereas our composites exceeded this value at 5 wt% GF loading.

**Figure 9:**
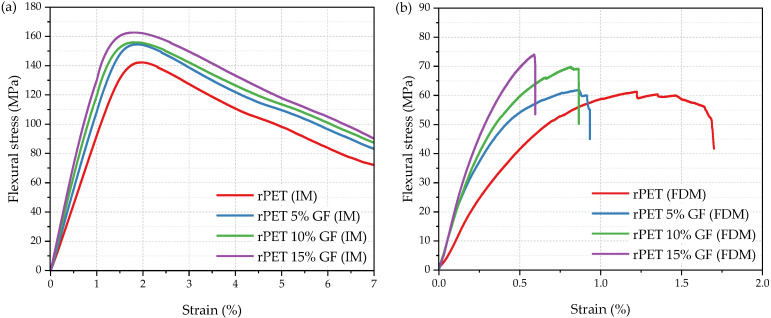
Representative flexural stress–strain curves of chain-extended rPET and rPET/glass fiber composites obtained via injection molding (**a**) and FDM 3D printing (**b**).

As in the tensile test discussed above, the manufacturing method was the dominant factor affecting the flexural properties of the specimens (Figure 9a,b, Table 6). Regarding the influence of fiber content on the flexural performance of the injection-molded samples, the reinforcing effect of the GF was evident. The flexural modulus (E_F_) steadily increased with increasing GF loading, reaching 4.5 GPa at 15% GF loading, which corresponds to a 25% improvement over the 3.6 GPa value of chain-extended rPET. Increasing the fiber content from 0 to 15 wt% resulted in a small increase in flexural strength (σ_F_), from 143.2 MPa to 161.9 MPa, accompanied by a minor decrease in flexural strain (ε_F_), from 2.0 to 1.7%. Although fabrication via FDM results in a lower ultimate flexural strength, the observed mechanical behavior patterns correlate strongly with those of conventionally injection-molded samples. Within the 3D-printed series, E_F_ and σ_F_ values generally increased with increasing GF loading. The E_F_ ranged from 1.0 GPa for rPET (FDM) to 2.7 GPa for rPET 15% GF (FDM), corresponding to approximately 28% of the value for rPET (IM) and approximately 60% of the value for rPET 15% GF (IM). It can be observed that σ_F_ is proportional to the contribution of the GF to the composite material. σ_F_ regularly increased from 61.7 to 74.2, as the weight fraction of GF increased from 5 to 15%. The maximum ε_F_ for the 3D-printed composite specimens was below 1%, further emphasizing their brittle character.

### 3.5. Impact Properties

**Figure 10:**
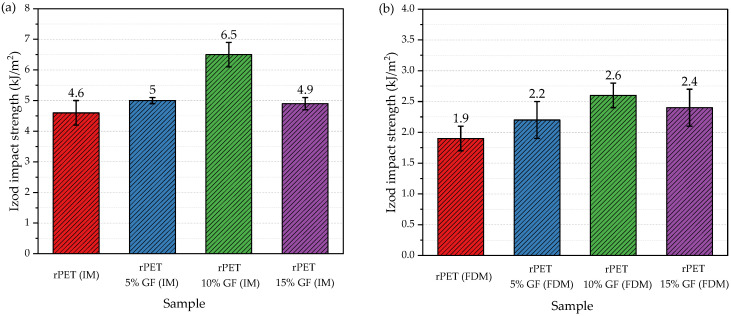
Izod impact strength of rPET and rPET/glass fiber composites; specimens obtained via injection molding (**a**) and FDM 3D printing (**b**).

Finally, the notched Izod impact properties were evaluated for both the injection-molded and FDM-manufactured samples (Figure 10a,b). The edgewise impact strength of the injection-molded rPET was 4.6 kJ/m^2^, which is slightly higher than that reported in the literature for virgin PET resin tested under the same conditions (3.8 kJ/m^2^) [49].

Transitioning to the FDM fabrication technique led to a pronounced decrease in the energy absorption of rPET, from 4.6 kJ/m^2^ to 1.9 kJ/m^2^, corresponding to more than a two-fold reduction in impact toughness. Nevertheless, the reported value is markedly higher than that of rPET (0.7 kJ/m^2^) and only slightly lower than that of rPET modified with E-EA-GMA and EEA impact modifiers (2.5 kJ/m^2^), both investigated by Rashwan et al. [25]. As shown in Figure 10, the specific energy absorbed by the material at fracture was slightly influenced by the addition of the GF reinforcement. The impact strength of the injection-molded (IM) composite specimens varied between 4.9 and 6.5 kJ/m^2^, with the 10 wt% glass fiber-reinforced rPET system exhibiting the maximum value. A similar trend was observed in the FDM-processed counterparts, although the recorded values were significantly lower, ranging from 2.2 to 2.6 kJ/m^2^.

**Figure 11:**
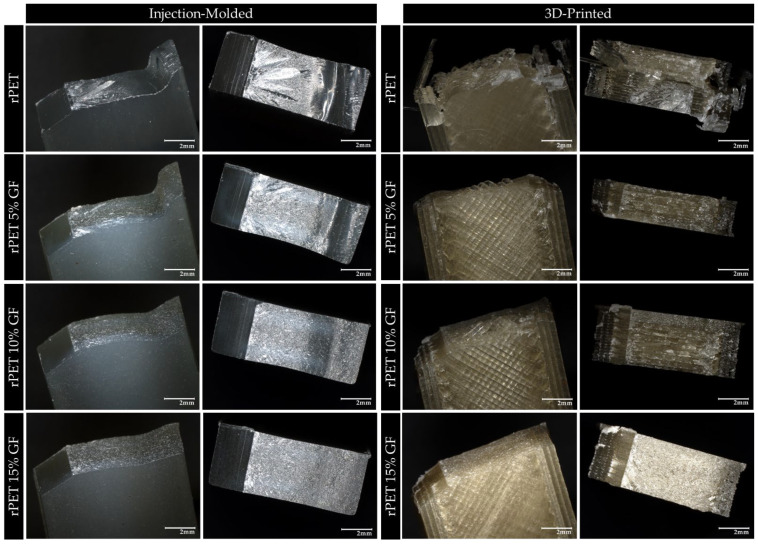
Fracture surfaces after Izod testing for injection-molded (**left panel**) and 3D-printed (**right panel**) specimens at 30× magnification.

Microscopic views of the fracture surfaces after the Izod impact testing of the injection-molded and 3D-printed specimens are presented in Figure 11. Light microscopy analysis of the injection-molded samples revealed a predominantly brittle fracture mechanism characterized by localized crazing near the notch root. Glass fiber reinforcement progressively shifts the failure mode from a single, fast crack propagating through a homogeneous matrix to a more tortuous, multi-event fracture process driven by crack deflection and fiber–matrix debonding. Nevertheless, no gross ductile deformation was observed, and the absence of significant shear lips or fibrous drawing was attributed to the low aspect ratio of the short glass fibers, which limited their capacity to induce large-scale plastic energy dissipation.

For the FDM-printed specimens, the fracture surfaces revealed a distinctly different failure mechanism governed by the layer-by-layer deposition architecture. Crack propagation occurs preferentially along interlayer boundaries rather than through the bulk of individual printed roads, making interlayer delamination the dominant failure mode in FDM. Voids between adjacent strands and between successive Z-layers, which are inherent to the FDM process, act as stress concentrators and pre-existing defect sites, severely limiting the impact resistance. However, with increasing GF content, a progressive reduction in layer delamination and fewer open voids between the parallel paths were observed. At the highest GF loading (15 wt%), the fracture surface appears more cohesive: while the layered structure remains detectable, the pronounced stepped delamination planes characteristic of lower GF loadings become less dominant, suggesting that fiber bridging across interlayer interfaces partially suppresses delamination and redirects fracture energy toward fiber pullout and debonding mechanisms.

## 4. Conclusions

In this study, short glass fiber-reinforced recycled polyethylene terephthalate composites were successfully prepared and processed for the first time. The proposed two-step chain extension strategy using an epoxy chain extender masterbatch effectively extended polyester chains via epoxy ring-opening reactions, yielding a high-molecular-weight PET recyclate. The latter is a viable matrix candidate for producing GF-reinforced composites with favorable end-use properties, suitable for melt-extrusion 3D printing and high-pressure injection molding.

A key advantage of the rPET systems investigated herein is the delayed crystallization kinetics, an effect that is especially pronounced in the fiber-reinforced systems. This phenomenon was corroborated by an increase in the half-crystallization times and a corresponding reduction in the Jeziorny rate constant, both indicating a slower overall crystallization rate in the rPET/GF composites. Moreover, it is important to note that the incorporation of GF significantly influences the crystallization mode of rPET. By limiting the spatial dimensions available for crystal development, glass fibers induce a transition from the dominant three-dimensional spherulitic growth observed in neat rPET to two-dimensional lamellar crystallization in rPET composites. This is particularly beneficial for MEX-AM applications, where semi-crystalline materials are rarely used as FDM feedstock, as the excessive crystallization leads to processing issues, such as shrinkage and warping. The mechanical test results showed that incorporating GF into rPET enhanced the tensile, flexural, and impact strength without markedly deteriorating the elongation or stiffness.

The study establishes a pathway for repurposing recycled PET bottles into functional 3D printing feedstock. The proposed approach offers a promising strategy for reducing the reliance on virgin monomers when formulating high-performance materials for additive manufacturing and other advanced applications. The combination of excellent processability, printability, and satisfactory mechanical performance underscores their strong potential for industrial-scale application. Future work will validate these materials in an industrial environment, with a particular focus on their application in hybrid manufacturing, specifically by combining large-format direct pellet extrusion (DPE) with subsequent part machining.

## Figures and Tables

**Table 1:** Printing and injection molding conditions.

Printing Parameters	Injection Molding Conditions
Nozzle temperature: 285 °C	Melt temperature: 280 °C
Layer thickness: 0.1 mm	Mold temperature: 30 °C
Build plate temperature: 90 °C	Injection speed: 35 mm/s
Raster angle: 45 °	Injection molding pressure: ~80 MPa
Printing speed: 60 mm/s	Holding pressure: ~40 MPa
Print cooling: 20%	Cooling time: 20 s

**Table 2:** Composition, basic physical and processing properties.

Samples	GF [wt%]	IV [dL/g]	MFR_285 °C, 2.16 kg_ [g/10 min]	d [g/cm^3^]	LoD [wt%]
rPET	0	0.97	10.7	1.31 ± 0.01	0.34
rPET 5% GF	5	0.94	8.3	1.34 ± 0.01	0.27
rPET 10% GF	10	0.93	5.8	1.38 ± 0.01	0.25
rPET 15% GF	15	0.93	3.4	1.41 ± 0.01	0.23

IV, intrinsic viscosity; MFR, melt flow rate measured at 285 °C with a 2.16 kg load; d, density; LoD, loss on drying.

**Table 3:** Thermal characteristics of chain-extended rPET and rPET/glass fiber composites.

Sample	*T_g_* [°C]	Δ*C_p_* [J/g·K]	*T_c_* [°C]	Δ*H_c_* [J/g]	*T_m_* [°C]	Δ*H_m_* [J/g]	*x_c_* [%]
rPET	78.3	0.20	185.0	32.9	232.1/238.4	32.9	16.5
rPET 5% GF	80.4	0.15	174.4	26.6	227.0	26.9	13.4
rPET 10% GF	79.8	0.16	173.4	25.5	226.0	25.9	12.9
rPET 15% GF	79.6	0.17	172.8	19.8	225.5	19.7	9.9

*T_g_*, glass transition temperature; Δ*C_p_*, specific heat increment of amorphous phase normalized by the polyester weight fraction and amorphous phase content; *T_c_*, Δ*H_c_*, temperature and enthalphy of crystallization, respectively; *T_m_*, Δ*H_m_*, temperature and enthalphy of melting, respectively; *x_c_*, degree of crystallinity.

**Table 4:** Crystallization parameters determined from DSC cooling scans and the results of Avrami/Avrami–Jeziorny analysis for rPET and rPET/GF composites under different cooling rates.

Sample	*R_c_* [°C/min]	*T*_0_ [°C]	*T_c_* [°C]	Δ*T* [J/g]	Δ*H_c_* [°C]	*t*_1/2_ [min]	*n*	*Z_t_* [min^−n^]	*Z_c_* [min^−n^]
rPET	2.5	196	188.3	91.7	35.2	5.02	2.36	0.02	0.21
	5	192.4	184.4	95.6	33.6	2.15	2.35	0.12	0.65
	7.5	190.4	182.3	97.7	31.6	1.44	2.31	0.30	0.85
	10	190.2	181.5	98.5	33.3	1.12	2.40	0.51	0.94
rPET 5% GF	2.5	181.2	168.0	112.0	28.5	6.28	2.08	0.01	0.16
	5	175.4	163.7	116.3	17.9	3.05	2.13	0.06	0.57
	7.5	174.3	162.7	117.3	17.4	2.15	2.19	0.13	0.76
	10	175.9	162.8	117.2	22.2	1.52	2.19	0.28	0.88
rPET 10% GF	2.5	180.6	167.4	112.6	21.9	6.32	2.02	0.01	0.15
	5	177	161.9	118.1	19.9	3.52	2.08	0.04	0.51
	7.5	175.6	161.1	118.9	18.2	2.25	2.09	0.11	0.74
	10	175.3	161.9	118.1	17.8	1.62	2.14	0.22	0.86
rPET 15% GF	2.5	176.4	161.9	118.1	13.4	6.99	2.02	0.00	0.12
	5	172.5	159.1	120.9	11.7	3.68	2.16	0.04	0.52
	7.5	173.8	158.7	121.3	11.1	2.57	2.10	0.07	0.70
	10	173.6	160.4	119.6	11.8	1.71	2.10	0.20	0.85

*R_c_*, cooling rate; *T*_0_, onset temperature; *T_c_*, crystallization peak temperature; Δ*T*, degree of supercooling; Δ*H_c_*, enthalpy of crystallization; *t*_1/2_, crystallization half-time; *n*, Avrami exponent; *Z_t_*, rate constant; *Z_c_*, Jeziorny modified rate constant.

**Table 5:** Thermomechanical properties of chain-extended rPET and rPET/glass fiber composites.

Sample	E′ at 25 °C (MPa)	T_α_ (°C)	tanδ at T_α_ (−)	T_α_′ (°C)	tanδ at T_α_′ (−)
rPET	2800	82.5	1.6	110.7	0.28
rPET 5% GF	3300	81.8	0.9	120.3	0.14
rPET 10% GF	3900	82.4	1.0	117.6	0.15
rPET 15% GF	4000	82.7	1.1	116.8	0.17

E′ at 25 °C, storage modulus at 25 °C; T_α_, DMTA representation of glass transition temperature; T_α_′, DMTA representation of glass transition temperature of amorphous domains restricted by the presence of crystalline domains, tanδ at T_α_—loss factor value at T_α_.

**Table 6:** Mechanical properties of chain-extended rPET and rPET/glass fiber composites determined for injection-molded (IM) and FDM 3D-printed (FDM) specimens.

Sample	E_T_ [GPa]	σ_y_ [MPa]	ε_y_ [%]	σ_b_ [MPa]	ε_b_ [%]	E_F_ [GPa]	σ_F_ [MPa]	ε_F_ [%]
Injection-Molded								
rPET (IM)	3.1 ± 0.2	65.4 ± 0.1	3.2 ± 0.1	45.9 ± 3.9	333.8 ± 27.4	3.6 ± 0.1	143.2 ± 1.3	2.0 ± 0.1
rPET 5% GF (IM)	3.3 ± 0.2	67.7 ± 1.1	3.2 ± 0.1	42.6 ± 2.4	43.3 ± 3.5	3.9 ± 0.2	151.2 ± 3.0	1.9 ± 0.1
rPET 10% GF (IM)	3.5 ± 0.1	71.9 ± 0.5	2.8 ± 0.1	44.5 ± 1.6	22.4 ± 1.6	4.0 ± 0.1	156.2 ± 0.2	1.8 ± 0.1
rPET 15% GF (IM)	4.4 ± 0.2	75.1 ± 1.8	2.7 ± 0.1	45.3 ± 2.2	15.8 ± 1.2	4.5 ± 0.1	161.9 ± 1.7	1.7 ± 0.1
3D-Printed								
rPET (FDM)	1.2 ± 0.2	-	-	36.4 ± 2.6	2.5 ± 0.1	1.0 ± 0.1	61.5 ± 2.9	1.4 ± 0.2
rPET 5% GF (FDM)	2.2 ± 0.1	-	-	43.5 ± 0.9	2.2 ± 0.2	2.5 ± 0.3	61.7 ± 1.3	0.9 ± 0.1
rPET 10% GF (FDM)	2.5 ± 0.1	-	-	47.9 ± 2.7	2.2 ± 0.3	2.5 ± 0.2	69.6 ± 3.3	0.8 ± 0.2
rPET 15% GF (FDM)	3.0 ± 0.2	-	-	46.2 ± 1.0	1.7 ± 0.3	2.7 ± 0.2	74.2 ± 3.4	0.6 ± 0.1

E_T_—tensile Young’s modulus; σ_y_ and ε_y_—tensile stress and elongation at yield, respectively; σ_b_ and ε_b_—tensile stress and elongation at break, respectively; E_F_—flexural Young’s modulus; σ_F_ and ε_F_—flexural stress and elongation at maximum stress, respectively.

## Data Availability

The original contributions presented in this study are included in the article/Supplementary Materials. Further inquiries can be directed to the corresponding author.

## Supplementary Materials

The following supporting information can be downloaded at: https://www.mdpi.com/article/10.3390/polym18101155/s1, Figure S1. DSC thermograms recorded during 1st heating of chain-extended rPET and rPET/glass fiber composites: specimens obtained via injection molding (a) and FDM 3D printing (b); Table S1. Thermal characteristics of chain-extended rPET and rPET/glass fiber composites. Values obtained from the 1st heating scan.
