# Supplementary material for: Short Glass Fiber-Reinforced Recycled Polyethylene Terephthalate Composites for Additive Manufacturing: Modification Strategies, Processing, Characterization and 3D Printing
Source: Polymers (Basel). 2026 May 8;18(10):1155. doi: 10.3390/polym18101155 (PMC13211043; doi:10.3390/polym18101155)
Supplement: Supplementary file 1 [file polymers-18-01155-s001.zip › polymers-4264879-supplementary.pdf]

Supplementary information

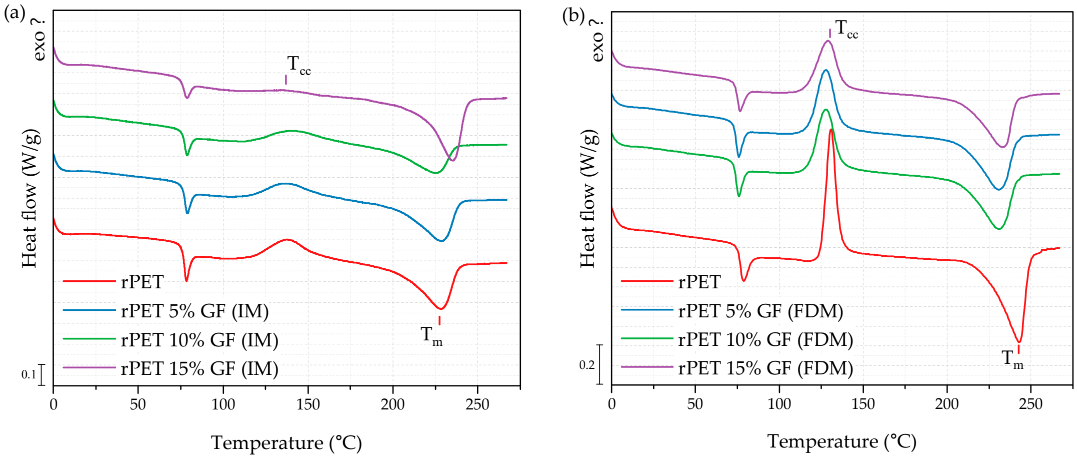

Figure S1. DSC thermograms recorded during 1st heating of chain-extended rPET and rPET/glass fiber composites: specimens obtained via injection molding (a) and FDM 3D printing (b).

Table S1. Thermal characteristics of chain-extended rPET and rPET/glass fiber composites. Values obtained from the 1st heating scan.

Sample	T_g [°C]	T_{cc} [°C]	ΔH_{cc} [J/g]	T_m [°C]	ΔH_m [J/g]
rPET	75.6	140.9	14.6	225.2	38.5
rPET 5% GF (IM)	75.7	136.7	17.8	228.9	31.5
rPET 10% GF (IM)	75.3	137.1	17.9	228.5	29.5
rPET 15% GF (IM)	74.5	138.6	8.1	235.3	20.5
rPET	75.3	130.9	27.5	240.0	54.8
rPET 5% GF (FDM)	73.7	129.6	28.9	229.2	39.8
rPET 10% GF (FDM)	72.6	127.5	26.9	231.0	38.4
rPET 15% GF (FDM)	73.2	129.2	27.4	233.1	36.4

T_g, glass transition temperature; T_{cc}, ΔH_{cc}, temperature and enthalphy of cold crystallization, respectively; T_m, ΔH_m, temperature and enthalphy of melting, respectively
